# Death burden of high systolic blood pressure in Sichuan Southwest China 1990–2030

**DOI:** 10.1186/s12889-020-8377-6

**Published:** 2020-03-29

**Authors:** Zhuo Wang, Yu Luo, Shujuan Yang, Mingliang Zuo, Rong Pei, Jun He, Yin Deng, Maigeng Zhou, Li Zhao, Hui Guo, Kun Zou

**Affiliations:** 1 Department of Chronic and Non-communicable Disease Control and Prevention, Sichuan Center of Disease Control and Prevention, Chengdu, China; 2 Key Laboratory of Birth Defects and Related Diseases of Women and Children (Sichuan University), Ministry of Education, West China Second University Hospital, Sichuan University, Chengdu, China; 3 West China School of Public Health and West China Fourth Hospital, Sichuan University, Chengdu, Sichuan China; 4 Department of Cardiovascular Ultrasound and Non-invasive Cardiology, Sichuan Provincial People’s Hospital, Affiliated Hospital of University of Electronic Science and Technology, Chengdu, Sichuan China; 5 School of Health Caring Industry, Sichuan University of Arts and Science, Dazhou, Sichuan China; 6 National Center for Chronic and Non-communicable Disease Control and Prevention, Chinese Center for Disease Control and Prevention, Beijing, China

**Keywords:** Death burden, High systolic blood pressure, Chronic diseases, Forecasting

## Abstract

**Background:**

Hypertension is highly prevalent and is the primary risk factor for cardiovascular disease (CVD) and chronic kidney disease (CKD). While declining in some developed countries, it is increasing rapidly in some developing countries. Sichuan province is the largest and underdeveloped region in southwest China, with 486 thousand square kilometers, more than 80 million residents, unbalanced economic development, and high prevalence, low awareness, low treatment and low control rate of hypertension. We forecasted the death burden due to high systolic blood pressure (SBP) in Sichuan from 1990 to 2030, to raise the awareness of public and government of the importance and benefits of hypertension control.

**Methods:**

We conducted secondary analysis based on data of Global Burden of Disease (GBD) 1990–2015, and predicted the population SBP level, population attributable fraction, and death burden for people aged 30–69 under different scenarios in 2030.

**Results:**

Comparing with natural trend, if the prevalence of high SBP can be reduced relatively by 25% by 2030, the deaths of non-communicable chronic diseases (NCDs), CVD and CKD would be reduced by 27.1 thousand, 26.2 thousand and 0.8 thousand for people aged 30–69; the mortality would be reduced by 10.8, 32.8 and 16.0%; and the premature mortality would be reduced by 9.9, 32.0 and 16.0%, respectively.

**Conclusions:**

Controlling or decreasing the prevalence of high SBP can significantly reduce the deaths, death rate and premature mortality of NCDs, CVD and CKD for the 30–69 years old population in Sichuan. There would be huge benefits for the governments to take cost-effective measures to control or reduce the prevalence of hypertension.

## Background

Hypertension is the primary risk factor for cardiovascular diseases (CVD) and nephropathy, significantly increasing the morbidity and mortality of heart attack, stroke, kidney failure, blindness etc. [1, 2]. High systolic blood pressure (SBP) ranks the first among risk factors and leads to 10.4 million deaths and 218 million disability-adjusted life-years (DALYs) in 2017 globally [3], which largely due to CVD [4–6]. While declining in developed countries, the prevalence of hypertension was increasing in developing countries in the last decade [7, 8]. The contribution from developing counties to the global disease burden of hypertension is increasing and the age of CVD onset is becoming younger [9, 10].

China, the largest developing country with one fifth of the global population, is facing similar problems, especially in its western regions where economy is relatively lagging behind [11, 12]. Studies showed that the prevalence of hypertension in these regions was higher, but the rate of awareness, treatment and control of hypertension in these underdeveloped region were lower than that of the developed regions in China. Moreover, compared with women, men had higher morbidity, lower awareness, treatment and control of hypertension [13–17].

Sichuan is the largest province in southwest China, with an area of 486 thousand square kilometers, and a population of more than 80 million, with multi-ethnics, different natural conditions, unbalanced economic development, rapid urbanization and demographic aging population. The prevalence of hypertension in Sichuan was 25.2%, similar to national average prevalence of 29.6%. However, the rates of awareness, treatment and control in Sichuan were 24.7, 14.7 and 3.7% respectively, much lower than the national average of 42.6, 34.1 and 9.3% in China [14, 18].

The prevention and control of hypertension not only depends on individuals, but also government policies to provide health prone public environment. Raising government awareness in these developing areas will benefit more people.

This study aimed to estimate the health benefits of hypertension control for the population especially labor force in developing regions, by assuming three changed scenarios based on population SBP level in 2015, which were natural trend, status unchanged and 25% decline of the prevalence of high SBP. And the death burden of high SBP aged 30–69 in Sichuan by 2030 was predicted, for that World Health Organization (WHO) reported many people died of chronic diseases in their 30s or 40s [19]. The evidence would provide more evidence of the benefits of hypertension prevention and control to support related health policy making by the governments.

## Methods

### Data collection

The data for prediction was provided by China Center for Chronic Disease Control and Prevention (CCDC) and Global Burden of Disease (GBD), which included mortality, population SBP, relative risks and number of people by age and gender from 1990 to 2015. GBD collaborations collected 114 published papers on national or local prevalence of high SBP in China to estimate prevalence in different regions by spatiotemporal Gauissian process regression (ST-GPR) [3, 20]. More information about GBD can be found in their home page [21]. The Sichuan population in 2030 was obtained from China Population and Development Research Center (CPDRC), which was estimated using dynamic cohort-component method [22].

### Comparative risk assessment (CRA)

CRA theory assumed that the exposure level of other independent risk factors remain unchanged except the one being analysed [3, 23]. We compared the exposure distribution of population SBP in Sichuan with the theoretical minimum risk exposure distribution or the counterfactual exposure distribution to estimate the proportion of the death burden in Sichuan due to high SBP. The counterfactual level of theoretical minimum risk exposure level (TMREL) of SBP was 110–115 mmHg, which found to be related to multiple cardiovascular and/or renal diseases when above it [24–26].

#### Three epidemic scenarios for high SBP in 2030

WHO required nine voluntary global targets. The target six was 25% relatively lower in the prevalence of high blood pressure than the current situation [27]. Based on that, we assumed three scenarios for prevalence of high blood pressure as follows:

- Unchanged (UN). Age and sex-specific SBP in 2030 were the same as in 2015.
- Natural trend (NT). Age and sex-specific SBP in 2030 was projected according to history change in 1990–2015.
- WHO target (WT). Age and sex-specific prevalence of high SBP in 2030 was reduced by 25% compared with the level in 2015.

### Estimation process

**Fig. 1:**
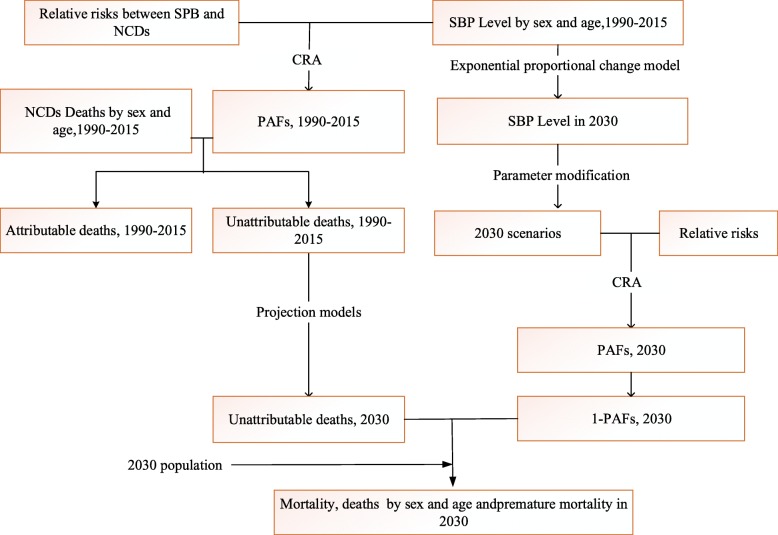
Analysis flowchart. SBP- systolic blood pressure; NCDs- non-communicable chronic diseases; PAFs- population attributable fraction; CRA- comparative risk assessment.

There were three main steps to project the death burden due to high SBP in 2030 as follows (Fig. 1). We used the total number of deaths, mortality, the probability of premature death of people aged 30–69, and their changes to reflect death burden of high SBP from 1990 to 2030. All analyses were performed using R software (R3.6.1).

- Estimating SBP distribution

Firstly, the data of SBP by sex and age in 1990–2015 were collected and organized. Secondly, SBP in natural trend by 2030 was deduced according to the exponential proportional change model, while the population SBP level in WHO target was estimated according to the reduced prevalence. Thirdly, SBP was corrected for regression dilution bias [28].

b. Estimating population attributable fraction (PAF)

Based on CRA and the correlation between NCDs mortality and SBP, PAF was calculated. It divided the number of NCDs deaths into two parts - attributable deaths of SBP and non-attributable deaths of SBP. Hypertension-related diseases mainly were CVD and CKD. Its formula was:

$$ PAF=\frac{\int_l^m RR(x)P(x) dx-{\int}_l^m RR(x){P}^{\prime }(x) dx}{\int_{x=0}^m RR(x)P(x) dx} $$

*x* was exposure level; P(x) was exposure distribution in population; *P*^′^(*x*) was TMREL; RR(x) was relative risk at SBP level x; l was minimum exposure level, while m was the maximum.

c. Estimating death burden

The calculated PAF was multiplied by the number of NCDs deaths, that is, the burden of NCDs caused by high blood pressure. Combined with the population composition in 2030, the crude mortality was further obtained, and premature mortality for people aged 30–69 which is globally comparable and independent of population composition was also obtained. The formula for premature mortality was:

$$ {}_{40}{q}_{30}=1-\underset{\mathrm{x}=30}{\overset{65}{\varPi }}\left(1{-}_5{q}_{\mathrm{x}}\right) $$

_40_q_30_was probability of death aged 30–69; _5_q_x_ was probability of death for each five-year age group [29].

### Ethical consideration

Our study is a secondary analysis of existing data from GBD. IRB Committee of Sichuan CDC hereby waives the need of ethical approval of research.

## Results

### Population SBP level

According to the past trend, regardless of age and sex, the average SBP level of the population would increase by years. The age and sex-specific SBP under WT scenario in 2030 was estimated by shifting the population normal distribution curve area to the left until meet the WHO’s target. It was estimated that the SBP of men under the age of 60 was higher than that of women, while that of women over 60 was higher than men. Meanwhile, the SBP increased with age. (Table 1).

**Table 1:** Age and sex-specific SBP level in 2015 and 2030 scenarios in Sichuan

Gender	Age	SBP level, mean (mmHg)^a^		
		2015/UN	NT	WT
Men	25~	124.2	134.9	122.2
	30~	123.2	131.3	121.3
	35~	125.6	134.6	123.5
	40~	128.3	138.9	125.9
	45~	129.6	140.3	127.0
	50~	133.4	143.0	130.3
	55~	136.9	144.7	133.2
	60~	137.0	144.0	133.2
	65~	141.9	148.7	137.0
	70~	141.0	144.7	136.3
	75~	143.3	146.3	138.0
	80~	143.5	150.1	138.1
Women	25~	116.4	124.4	115.0
	30~	117.0	124.1	115.7
	35~	119.2	126.9	117.7
	40~	123.9	131.2	122.0
	45~	128.0	138.2	125.6
	50~	130.7	138.3	128.0
	55~	134.9	143.4	131.5
	60~	138.8	145.0	134.6
	65~	142.4	148.2	137.3
	70~	144.3	149.0	138.6
	75~	142.9	147.4	137.7
	80~	145.8	150.6	139.6

^a^*UN* Unchanged Scenario; *NT* Natural trend Scenario; *WT* Reduced 25% Scenario

### Disease burden of deaths and mortality for 30–69 years old in 2030 under natural trend

The death burden of CVD, CKD and NCDs for people aged 30–69 under historical trend were presented in Table 2. The numbers of death and death rate in 2030 would be 250.2 thousand and 506.3 per 100 thousand for NCDs, 79.9 thousand and 161.8 per 100 thousand for CVD, and 5.2 thousand and 10.5 per 100 thousand for CKD. Compared with that of 2015, the death rate of NCDs, CVD and CKD would decrease, while the numbers of death of CVD and CKD would increase (Table 2, Additional file 1: Table S1 and Additional file 2: Figure S1).

**Table 2:** Deaths number, crude mortality, premature mortality and change of main NCDs for people aged 30–69 from 2015 to 2030 in Sichuan

Gender	Disease ^a^	2015			UN^b^			NT^b^			WT^b^			Reduction in deaths (in thousands)^c^		Change of mortality rate (%)^c^		Change of premature mortality (%)^c^	
		Deaths (thousands)	Death rate (per 100,000)	Premature mortality (%)	Deaths (thousands)	Death rate (per 100,000)	Premature mortality (%)	Deaths (thousands)	Death rate (per 100,000)	Premature mortality (%)	Deaths (thousands)	Death rate (per 100,000)	Premature mortality (%)	UN	WT	UN	WT	UN	WT
Both	NCDs	267.1	610.2	23.6	231.3	468.1	17.1	250.2	506.3	18.3	223.1	451.6	16.5	−18.9	−27.1	− 7.5	− 10.8	− 6.9	−9.9
	CVD	79.0	180.5	7.7	61.6	124.7	4.9	79.9	161.8	6.3	53.7	108.7	4.3	−18.3	−26.2	−22.9	−32.8	−22.2	− 32.0
	CKD	4.6	10.6	0.5	4.6	9.4	0.4	5.2	10.5	0.4	4.4	8.8	0.4	−0.6	−0.8	−11.0	−16.0	− 11.0	− 16.0
Men	NCDs	177.0	803.9	29.7	162.4	652.1	23.2	175.7	705.7	24.9	156.9	630.2	22.5	−13.4	−18.8	−7.6	−10.7	−6.7	−9.4
	CVD	50.2	228.0	9.6	40.8	163.9	6.5	53.8	216.1	8.4	35.5	142.7	5.7	−13.0	−18.3	−24.1	−34.0	−23.2	−32.9
	CKD	2.7	12.3	0.5	2.9	11.5	0.5	3.2	12.9	0.5	2.7	10.9	0.4	−0.4	−0.5	−11.1	−15.8	−11.1	−15.9
Women	NCDs	90.1	414.3	16.8	68.9	281.2	10.5	74.5	303.8	11.3	66.2	270.1	10.1	−5.5	−8.3	−7.4	−11.1	−7.0	−10.5
	CVD	28.8	132.5	5.8	20.8	84.9	3.3	26.1	106.5	4.2	18.2	74.2	2.9	−5.3	−7.9	−20.4	−30.4	−19.8	−29.8
	CKD	1.9	8.8	0.4	1.8	7.2	0.3	2.0	8.0	0.3	1.7	6.7	0.3	−0.2	−0.3	−10.8	−16.2	−10.8	−16.2

^a^*NCDs* non-communicable chronic diseases; *CVD* cardiovascular diseases; *CKD* chronic kidney disease

^b^*UN* Unchanged Scenario; *NT* Natural trend Scenario; *WT* Reduced 25% Scenario

^c^ Compared with natural trend scenarios

The death burden would be different by gender and disease. For men, the deaths of CVD would increase from 50.2 thousand in 2015 to 53.8 thousand in 2030, while the mortality rate would decrease from 228.0 per 100 thousand to 216.1 per 100 thousand. However, the deaths and mortality of CKD for men would both increase. For women, the deaths for CVD would decrease from 28.8 thousand in 2015 to 26.1 thousand in 2030, and the mortality rate would decrease from 132.5 per 100 thousand to 106.5 per 100 thousand, while the deaths of CKD would slightly rise but the mortality would decline. The deaths and mortality of NCDs in both genders would decline.

### Benefits for deaths and mortality aged 30–69 in different scenarios

Comparing with NT, the deaths of NCDs, CVD and CKD would further decline under the scenarios of UN or WT. In the scenarios of UN and WT, there would be 231.3 thousand and 223.1 thousand deaths, a decrease of 18.9 thousand and 27.1 thousand, respectively. Meanwhile, the mortality would be 468.1 per 100 thousand and 451.6 per 100 thousand, a decrease of 7.5 and 10.8%, respectively (Table 2).

Reducing the prevalence of high SBP would have different benefits for men and women. Comparing with NT, the deaths of CVD would be 40.8 thousand and 35.5 thousand in UN and WT, a decrease of 13.0 thousand and 18.3 thousand for men, respectively; while a decrease of 5.3 thousand and 7.9 thousand for women, respectively. For men, the mortality of CVD would be 163.9 per 100 thousand and 142.7 per 100 thousand, a decrease of 24.1 and 34.0%, which would be 20.4 and 30.4% for women. Similar changes were observed for CKD that the decline rate in women would be lower than that in men.

### Reduction of premature mortality for age 30–69 in different scenarios

The probability of dying for those aged 30–69 would be declining by years. The premature mortality of NCDs was 23.6% in 2015, and would be 18.3, 17.1 and 165.5% in scenarios of NT, UN and WT by 2030. For CVD, the numbers would be 7.7, 6.3, 4.9, and 4.3%, respectively; and for CKD, 0.5, 0.4, 0.4 and 0.4%, respectively.

For each disease, men would have generally higher premature mortality than women, while the decline rate of premature mortality would be different by sex. Comparing with NT, the premature mortality of CVD in WT would drop the most by 32.0%, with 32.9% for male and 29.8% for female; while that of CKD would be 16.0%, with 15.9% for male and 16.2% for female (Table 2).

**Fig. 2:**
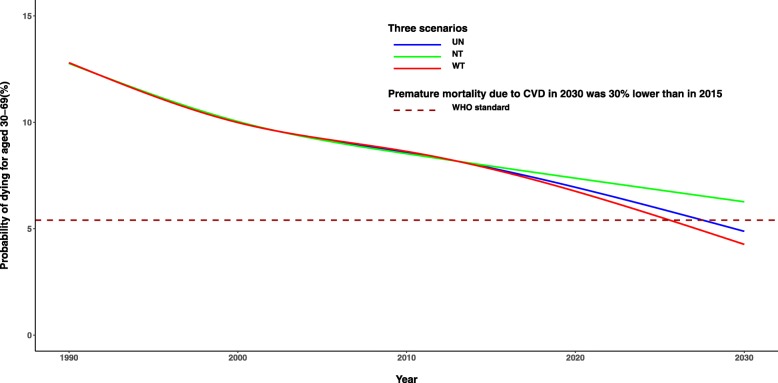
Probability of premature death due to CVD for aged 30-69 in Sichuan from 1990 to 2030 in Sichuan. CVD-cardiovascular diseases; UN-Unchanged Scenario; NT- Nature trend Scenario; WT- Reduced 25% Scenario.

We took the WHO target of 30% reduction in premature mortality from CVD by 2030 as standard (Fig. 2), which was claimed in “2030 Agenda for Sustainable Development” and was 5.4% in Sichuan [30]. If we can keep the prevalence of high SBP in 2030 unchanged as in 2015, the premature mortality for age 30–69 would be 4.9%. If we can further reduce the prevalence by 25%, it would be 4.3%.

## Discussion

The impact of hypertension on NCDs has been recognized for more than 60 years. Evidence showed that when the SBP exceeded 115 mmHg, the incidences for stroke and coronary heart disease began to rise, regardless of the inconsistent criteria for hypertension [31–33]. Based on these evidence, we projected death burden related to high SBP in Sichuan, the largest developing province in Southwest China. Comparing with the natural trend in 2030, if the prevalence of high SBP could be reduced by 25%, the deaths of NCDs, CVD and CKD would reduce by 27.1 thousand, 26.2 thousand and 0.8 thousand for people aged 30–69; and the mortality of the three types of diseases would reduce by 10.8, 32.8 and 16.0%; and the premature mortality would reduce by 9.9, 32.0 and 16.0%, respectively.

WHO reported that the prevalence of hypertension in developed countries was declining, but rising rapidly in developing countries [7]. In our study, we found that the population SBP level in Sichuan would be continuously rising, while the death rates of NCDs, CVD and CKD would be declining, which was similar to the national trend [34]. It’s good to see that there would be generally downward trend of deaths, mortality and probability of dying for people aged 30–69. This may be attributed to the improvement of economic, which is closely related to progress of public health services and mortality [4, 35]. While the death number for CVD and CKD would increase mainly due to aging population and increasing of population size, which can be proved by the reducing of premature mortality.

Although the NCDs mortality continued to decline, the high and rising prevalence of high SBP, low awareness and control of hypertension, aging population may bring a large number of non-fatal disease burden in the future [10, 36]. If we ignore this existing circumstances, it will become an obstacle to achieve the “healthy China action plan” and WHO requirement, who demand the standardized management rate of hypertension would be no less than 60 and 70% in 2022 and 2030 respectively, and the premature mortality of CVD would be reduced by 30% [27, 30, 37].

If we can maintain the current prevalence of high SBP and steadily improve our economic and medical circumstance, we would achieve the WHO demand of reducing premature mortality due to CVD. But we need to do more if we want to make sure that our goals can be met or achieved earlier. This study showed the health benefits under different scenarios, which provided a clear vision to government for decision-making. It highlighted the importance of hypertension control for NCDs, CVD and CKD, and also found that men would benefit more than women.

Compared with the areas with relatively good economic conditions in the east and central China, there is still a lot of room for progress in the west regions, while number of hypertension patients under standardized management in the east and central was twice that in the west [38, 39], and management rate of hypertension patients in Sichuan Province was about 40% [40].

There are many international experiences and evident measures in prevention and control of hypertension, such as comprehensive intervention strategies, lifestyle changes and drug therapy [41, 42]. We also hope to emphasize that different regions should choose cost-effective measures based on local conditions. For example, based on the situation in Sichuan, improvements of the following five aspects may be effective. Firstly, we can rely on National Essential Public Health Services (EPHS), which can improve the treatment and control on patients, as well as basic healthcare services to every citizen [43, 44].

Secondly, it is particularly important to take care of the elderly as the population, is aging. Research found that the hypertension control rate was especially low in the elderly [45]. The elderly usually suffered from multiple diseases. Hypertension not only coexisted with common chronic diseases such as diabetes, but also increased the risk of other diseases [46].

Thirdly, providing essential free medicines to the patients in need, especially for the poor. The economic development and distribution of medical resources in Sichuan was unbalanced, and so was the primary care system. If the EPHS or other policies can provide long-term free essential treatments, it may improve hypertension control, especially in the poorer areas.

Fourthly, salt reduction actions. There are many risk factors of hypertension, including personal behaviors and environmental factors [47]. Sichuan dishes is famous for its flavour deep and rich taste, but the high sodium problem is common [48–50]. Thus, salt reduction would be the most effective way to reduce blood pressure in the population, which is one of the action strategies recommended by the WHO.

Lastly, the WHO CVD risk charts can be a useful tool to classify the risk of the population and help to design the tailored control strategies [51], which may effectively save the limited medical resources including financial and human resources, especially in underdeveloped areas with large population.

There are few strengths of this study. Firstly, it may be one among few studies to predict the death burden of high HBP toward 2030 and potential health benefits of hypertension control for the large poorer region of China. Secondly, the GBD and WHO study data and methods were adopted in this study, which strengthened the methodological rigorous and international comparability of this study. Other developing countries or regions with similar contexts may adopt the methods and make international or regional comparison with findings from our study to support their health policy making. Thirdly, the comprehensive quantitative prediction and presentation of the death burden NCDs, CVD and CKD of the working age group (30–69) and the benefits of control measures under different scenarios provided governments and the public explicit evidence to empower health communication of hypertension control and support relevant policy making.

There were several limitations of this study. Firstly, the estimation was based on China GBD data. Although the validation of data collecting and prevalence estimation methods were published in other literatures [3, 5]. Its accuracy still could be improved by combining with local survey data in the future. Secondly, this study assumed that all factors except SBP followed natural trend, so many other disease-related variables were not included in the prediction, such as economic, behavioral, environmental factors etc. Further studies would be enhanced to take more risk factors into account to obtain more precise estimation.

## Conclusions

There is good potential and would be huge health benefits for hypertension control actions in Sichuan China. If the prevalence of high SBP can be reduced by 25% at 2030 on the 2015 basis, there would be 27.1 thousand less deaths of NCDs, and a reduction of 10.8% of death rate and 9.9% of premature mortality for people aged 30–69 compared with the natural trend; and similar for the CVDs and CKD. It’s time for governments to take cost-effective measures to control hypertension according to local conditions and reduce disease burden of hypertension in the next few years.

## Supplementary information

**Additional file 1.** Deaths number, crude mortality, premature mortality of main NCDs for people aged 30–69 from 1990 to 2010 in Sichuan. The table comprises the indicators and changes of death burden in Sichuan Province from 1990 to 2000.

**Additional file 2.** Death rate of CVD for people aged 30–69 from 1990 to 2030 in Sichuan. The figure comprises the changes of premature mortality due to cardiovascular diseases in Sichuan Province from 1990 to 2030 under three scenarios.

## Data Availability

The data that support our study are available from Sichuan CDC and CCDC but restrictions apply to the availability of these data. National data can be accessed from the following link in public: https://gbd2016.healthdata.org/gbd-search/, but Provincial data were not available in public. Data are however available from the authors on reasonable request.

## Abbreviations

CCDC: China Center for Chronic Disease Control and Prevention
CKD: Chronic kidney disease
CPDRC: China Population and Development Research Center
CRA: Comparative Risk Assessment
CVD: Cardiovascular disease
DALYs: Disability-adjusted life-years
EPHS: Essential Public Health Services
GBD: Global Burden of Disease Study
NCDs: Non-communicable chronic diseases
NT: Natural trend
PAF: Estimating population attributable fraction
RR: Relative risk
SBP: Systolic blood pressure
ST-GPR: Gauissian process regression
TMREL: Theoretical minimum risk exposure level
UN: Unchanged
WHO: World health organization
WT: WHO target
