# Supplementary material for: Death burden of high systolic blood pressure in Sichuan Southwest China 1990–2030
Source: BMC Public Health. 2020 Mar 29;20:406. doi: 10.1186/s12889-020-8377-6 (PMC7104502; doi:10.1186/s12889-020-8377-6)
Supplement: Supplementary file 1 — Additional file 1. Deaths number, crude mortality, premature mortality of main NCDs for people aged 30–69 from 1990 to 2010 in Sichuan. The table comprises the indicators and changes of death burden in Sichuan Province from 1990 to 2000. [file 12889_2020_8377_MOESM1_ESM.docx]

**Supplemental materials**

Additional file 1. Additional tables.

Table S1. Deaths number, crude mortality, premature mortality of main NCDs for people aged 30-69 from 1990 to 2010 in Sichuan

| Gender | Disease ^a^ | 1990 | | | 1995 | | | 2000 | | | 2005 | | | 2010 | | |
| --- | --- | --- | --- | --- | --- | --- | --- | --- | --- | --- | --- | --- | --- | --- | --- | --- |
|  |  | Deaths (thousands) | Death rate (per 100,000) | Premature mortality (%) | Deaths (thousands) | Death rate (per 100,000) | Premature mortality (%) | Deaths (thousands) | Death rate (per 100,000) | Premature mortality (%) | Deaths (thousands) | Death rate (per 100,000) | Premature mortality (%) | Deaths (thousands) | Death rate (per 100,000) | Premature mortality (%) |
| Both | NCDs | 321.2 | 788.7 | 37.4 | 290.4 | 723.3 | 34.4 | 267.2 | 670.2 | 32.7 | 269.4 | 631.1 | 30.0 | 253.1 | 577.8 | 25.6 |
|  | CVD | 93.1 | 228.7 | 13.0 | 77.0 | 191.8 | 10.8 | 69.8 | 175.0 | 10.1 | 73.8 | 172.8 | 9.5 | 74.3 | 169.7 | 8.5 |
|  | CKD | 4.7 | 11.6 | 0.6 | 4.3 | 10.8 | 0.6 | 4.4 | 11.0 | 0.6 | 4.6 | 10.8 | 0.6 | 4.5 | 10.4 | 0.5 |
| Men | NCDs | 187.5 | 875.9 | 40.9 | 174.9 | 834.4 | 38.5 | 163.2 | 792.3 | 37.1 | 169.2 | 773.6 | 34.9 | 165.1 | 743.0 | 31.4 |
|  | CVD | 51.2 | 239.0 | 13.8 | 44.0 | 209.8 | 11.8 | 40.1 | 194.7 | 11.1 | 43.9 | 200.5 | 10.9 | 46.1 | 207.5 | 10.2 |
|  | CKD | 2.5 | 11.6 | 0.6 | 2.3 | 11.1 | 0.6 | 2.4 | 11.6 | 0.6 | 2.6 | 11.7 | 0.6 | 2.6 | 11.8 | 0.6 |
| Women | NCDs | 133.7 | 692.0 | 33.5 | 115.5 | 602.0 | 29.6 | 104.0 | 539.7 | 27.6 | 100.2 | 481.4 | 24.2 | 88.0 | 407.6 | 19.1 |
|  | CVD | 42.0 | 217.2 | 12.2 | 33.0 | 172.2 | 9.6 | 29.7 | 154.0 | 8.9 | 29.9 | 143.7 | 8.1 | 28.2 | 130.8 | 6.8 |
|  | CKD | 2.2 | 11.6 | 0.6 | 2.0 | 10.4 | 0.5 | 2.0 | 10.4 | 0.6 | 2.0 | 9.8 | 0.5 | 1.9 | 8.9 | 0.4 |

a NCDs-non-communicable chronic diseases; CVD-cardiovascular diseases; CKD-chronic kidney disease.
