# Supplementary figures and images for: Death burden of high systolic blood pressure in Sichuan Southwest China 1990–2030
Source: BMC Public Health. 2020 Mar 29;20:406. doi: 10.1186/s12889-020-8377-6 (PMC7104502; doi:10.1186/s12889-020-8377-6)

Death rate of CVD for people aged 30–69(per 100,000)

Three scenarios

UN  
NT  
WT

1990

2000

2010

2020

2030

Year

250  
200  
150  
100

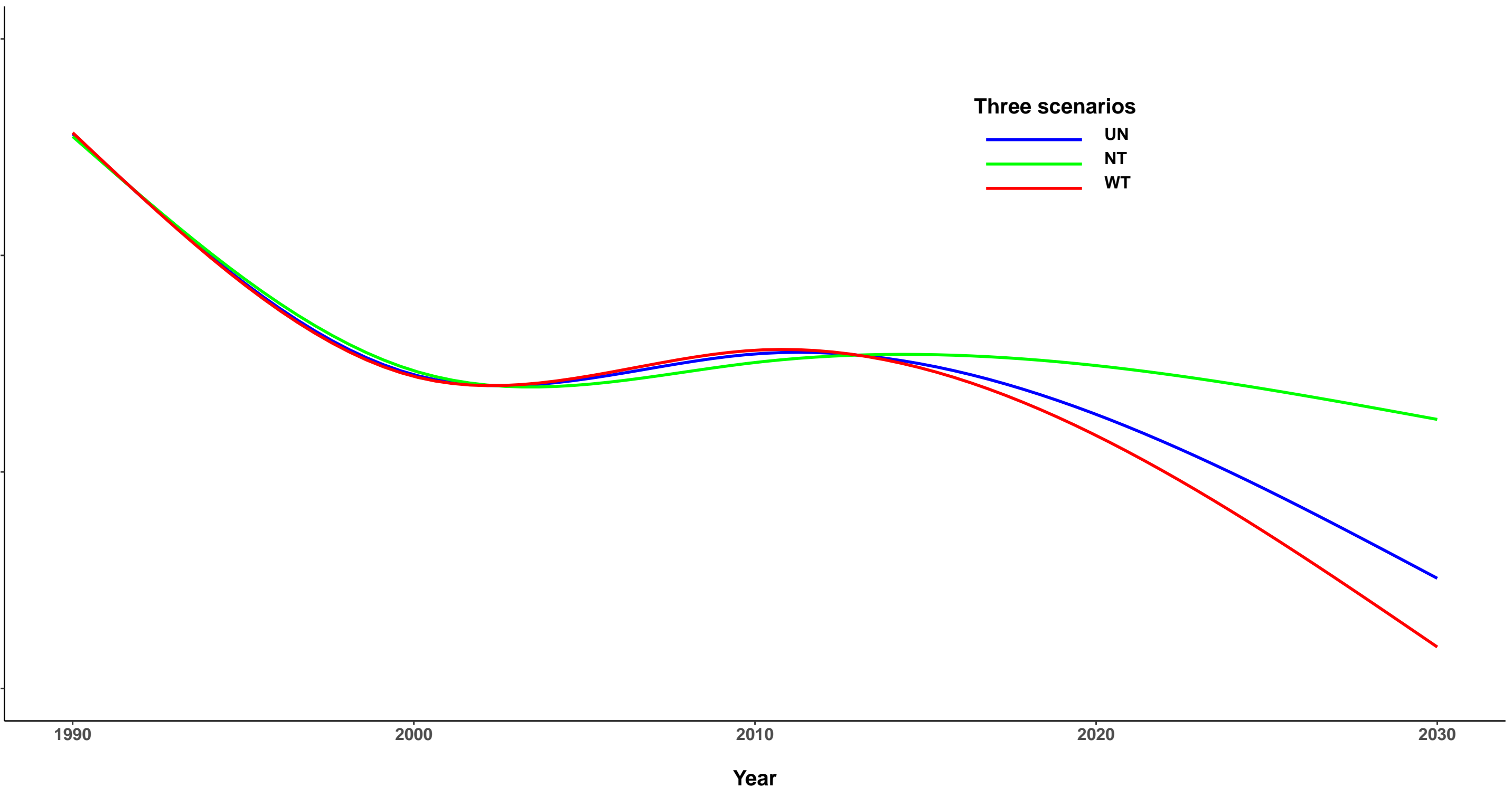

Supplement: Supplementary file 2 — Additional file 2. Death rate of CVD for people aged 30–69 from 1990 to 2030 in Sichuan. The figure comprises the changes of premature mortality due to cardiovascular diseases in Sichuan Province from 1990 to 2030 under three scenarios. [file 12889_2020_8377_MOESM2_ESM.pdf]
